# Unraveling the genetic and molecular mechanisms of anthocyanin biosynthesis and accumulation in maize kernels

**DOI:** 10.3389/fpls.2026.1771678

**Published:** 2026-02-11

**Authors:** Mengting He, Jiansheng Li, Weiwei Jin, Jingyan Liu

**Affiliations:** 1 Tianjin Key Laboratory of Intelligent Breeding of Major Crops, College of Agronomy & Resources and Environment, Tianjin Agricultural University, Tianjin, China; 2 State Key Laboratory of Maize Bio-breeding, National Maize Improvement Center, China Agricultural University, Beijing, China

**Keywords:** anthocyanin biosynthesis, fresh corn, maize kernels, regulation pathway, stress response

## Abstract

Anthocyanins are a class of water-soluble flavonoid pigments found in maize (*Zea mays* L.) kernels that exhibit strong antioxidant, anti-inflammatory, and anticancer properties, enhancing both the nutritional value and stress resilience of maize. Recent researches have made significant progress in elucidating the molecular mechanisms underlying anthocyanin accumulation in maize kernels. This review summarizes current knowledge on the genetic and molecular regulation of anthocyanin biosynthesis in maize kernels, highlighting the roles of structural and regulatory genes and the central MBW (MYB-bHLH-WD40) complex. It also integrates emerging insights into transcriptional regulation, signaling pathways, and stress-responsive mechanisms that collectively modulate anthocyanin accumulation in maize. These findings establish a coherent conceptual framework to guide future research and to facilitate the rational, targeted breeding of maize varieties enriched in anthocyanins. Overall, this review provides a solid theoretical foundation to support molecular breeding strategies aimed at developing anthocyanin-rich maize and to advance the industrial development and application of functional fresh maize.

## 1 Introduction

As one of the world’s most important cereal crops, maize (*Zea mays* L.) plays a crucial role in global food security, industrial production, and human nutrition. Beyond serving as a major food source, maize also has a wide range of industrial applications and holds substantial economic value. It is extensively utilized across multiple sectors, such as in food production, livestock feed, as a vegetable, in snack manufacturing, and as a key industrial raw material. Additionally, maize constitutes the primary component of animal feed, representing the largest share of the global feed market. In China, maize ranks as the third most important cereal crop after rice and wheat, making a significant contribution to the nation’s overall grain yield. In recent years, China’s fresh corn industry has experienced rapid growth, marked by a swift expansion in cultivation areas. Fresh corn has emerged as a major contributor to the maize industry, following feed maize and maize deep processing. Currently, the cultivation area for corn has reached about 1.67 million hectares, representing an astonishing 500-fold increase over the past four decades. More than 500 processing enterprises are now involved in the fresh corn industry chain, contributing to a total output value of around 25 billion yuan. This remarkable growth has positioned China as the world’s largest producer and consumer of fresh corn (Li et al., 2025a). In 2024, China’s maize planting area expanded to 44.74 million hectares, an increase of 522,000 hectares (a 1.2% increase) compared with 2023. During the same year, total maize production reached 294.915 billion kilogram up 6.075 billion kilogram from the previous year, reflecting a 2.1% rise. Maize accounted for 41.7% of China’s total grain output, and by 2025, the overall maize market is expected to reach a value of approximately 577 billion yuan. The cultivation of fresh-eating maize offers significantly greater economic returns than those of traditional grain crops, highlighting its enormous market potential and promising prospects for future growth.

Fresh corn refers to maize harvested during the milk stage for direct consumption or processing, and it primarily includes sweet corn, waxy corn, sweet-waxy corn, and baby corn (Li et al., 2022b). Fresh corn kernels are rich in a wide range of nutrients. In particular, sweet corn contains about 5–10 times more sugar than ordinary corn, which is a type of corn mainly used for feed, industrial raw materials or processed grain (Usman et al., 2024); (Yu and Moon, 2021), and its protein content exceeds 13% (Mushtaq et al., 2025). Additionally, sweet corn is rich in natural vitamins, including A and E, as well as B-complex vitamins such as B_1_ and B_2_ (Feng et al., 2020; Xie et al., 2017). These nutrients play important roles in promoting cell division, delaying the aging process, and supporting overall physiological regulation and health. In waxy maize endosperm, amylopectin constitutes the majority of the starch, and its digestibility is about 10% higher than that of ordinary corn (Yangcheng et al., 2013). Baby corn also possesses high nutritional value, containing essential minerals like calcium, phosphorus, and iron, making it an excellent choice for a healthy diet (Singh et al., 2014). Additionally, maize oil, extracted from maize kernels, is composed of more than 80% unsaturated fatty acids and is rich in functional compounds such as vitamins E and A, as well as lecithin (Zhao et al., 2021). These compounds help reduce cholesterol levels and aid in the prevention of obesity and cardiovascular diseases (Zhao et al., 2021). Anthocyanins are naturally occurring, water-soluble pigments responsible for the red, blue, or purple colors seen in many plants (Khoo et al., 2017). They are commonly found in fruits, vegetables, and flowers and belong to the flavonoid subclass of polyphenolic compounds. Anthocyanins are known for a range of beneficial biological activities, including antioxidant, anti-inflammatory, and anticancer effects (Aboonabi et al., 2020; Chen et al., 2014; De Sousa Moraes et al., 2019). Researches indicate that they may also help prevent chronic diseases such as cardiovascular diseases and diabetes (Dong et al., 2022; Ghosh and Konishi, 2007). In maize, especially in purple and black varieties, anthocyanin levels are relatively high. These varieties can be consumed directly or used as raw materials for extracting anthocyanins. They are popular in the market because of their distinctive coloration, coupled with high nutritional value and health benefits. Additionally, they have broad potential applications in the food, pharmaceutical, and cosmetic industries. As a result, researches into the genetic and molecular mechanisms of anthocyanin biosynthesis in maize has been steadily advancing. At the same time, breeding and cultivation techniques for anthocyanin-rich maize are being continuously refined to increase both yield and anthocyanin content, ensuring they meet growing market demand (Paulsmeyer et al., 2022). With the advancement of modern agriculture, nutritional security has gained increasing attention, expanding beyond the traditional focus on food safety to encompass a broader concept that includes both food and nutritional safety. Fresh corn, rich in essential nutrients vital for human health, stands out as an excellent candidate for nutritional fortification due to its shorter cooking and processing time, which helps minimize nutrient loss. Owing to its high nutritional value and diverse applications, fresh corn plays a key role in the ongoing transition from ensuring food safety alone to achieving comprehensive food and nutritional security. In modern agriculture, fresh corn serves as an ideal crop for biofortification, not only because of its rich nutritional profile but also due to its potential for further enhancement through biofortification and genetic breeding. These attributes make it a valuable contributor to improving food and nutritional security.

The biosynthesis and accumulation of anthocyanins in maize kernels play a critical role in determining both grain color and nutritional composition, providing a foundation for the breeding of high-value.

cultivars (Hong et al., 2020). The variation in maize kernel color primarily results from two major pigment classes: anthocyanins and carotenoids (Di Gioia et al., 2020). Anthocyanins are mainly responsible for producing dark colors such as purple and red, while carotenoids generate yellow tones. Differences in the relative amounts and types of these two pigments create the wide spectrum of colors observed in maize, from purple to red to yellow. This vivid coloration not only enhances the visual appeal of maize but also enriches its nutritional value, providing essential health benefits to consumers. The biosynthesis of anthocyanins in maize is a complex biochemical process that involves multiple enzymes and gene regulatory mechanisms. It starts with phenylalanine and progresses through a series of enzymatic reactions, ultimately producing anthocyanins (Springob et al., 2003). This pathway is tightly regulated by transcription factors (TFs), including MYB, bHLH, and WD40 (Carpi et al., 2023). Studies have demonstrated that these three TFs form a complex that acts as the central regulatory module controlling anthocyanin biosynthesis in maize. This complex regulates the expression of downstream structural genes, orchestrating the entire biosynthetic process of anthocyanins (Qin et al., 2021). Anthocyanins not only provide plants with antioxidant activity and protection against ultraviolet (UV) radiation but also play important roles in mitigating environmental stresses such as low temperature and salinity. In maize, the *ZmBZ1* gene is critical for anthocyanin accumulation in kernels. The glycosyltransferase encoded by *ZmBZ1* enhances maize tolerance to salt stress by facilitating anthocyanin biosynthesis under saline conditions (Wang et al., 2022a). Light is another important factor regulating anthocyanin accumulation. In purple waxy maize, light exposure significantly increases kernel anthocyanin content by upregulating the expression of key anthocyanin biosynthetic genes, including *F3H* and *UGT* (Lu et al., 2023). Anthocyanins are potent antioxidants that exhibit stronger free radical scavenging activity in purple corn than conventional antioxidants like butylated anisole (BHA), vitamin E, catechin, and quercetin (Blaner et al., 2021; Brewer and Safety, 2011; Coșarcă et al., 2019; Felter et al., 2021; Xu et al., 2019). By modulating anthocyanin accumulation in maize kernels, it is possible to produce fresh corn products enriched with bioactive compounds, creating functional foods with potential health benefits for human consumption (Cai et al., 2023). Currently, researches on maize anthocyanins has largely concentrated on the biosynthetic pathway of anthocyanins. However, there is limited understanding of the genetic characteristics of anthocyanin-related genes and the influence of environmental factors on anthocyanin synthesis. Future studies could investigate the cross-regulatory mechanisms between anthocyanin biosynthesis and other metabolic pathways, such as carotenoid and polyphenol metabolism. Additionally, exploring the role of epigenetic modifications, including DNA methylation and histone modification, in anthocyanin regulation could provide a more comprehensive insight into its complex regulatory network.

## 2 Fundamental characteristics of anthocyanins in maize kernels

Anthocyanins are a class of flavonoid compounds and represent one of the most common types of plant secondary metabolites (Cao et al., 2020). These natural, water-soluble pigments are widely distributed throughout various plant tissues. Structurally, anthocyanins share a core 2-phenylbenzopyrylium (C6-C3-C6) skeleton, and their diversity mainly stems from variations in the substituent groups at the R1, R2, and R3 positions on the B-ring (Xie et al., 2024). These structural differences give rise to several major types, including cyanidin (Cy), pelargonidin (Pg), malvidin (Mv), delphinidin (Dp), peonidin (Pn), and petunidin (Pt). In maize kernels, anthocyanins exhibit considerable complexity in both form and classification. They are predominantly localized in the aleurone layer, scutellum, and pericarp tissues. Both cyanidin and pelargonidin pigments are present in corn kernels, but their contents depend on the specific variety and color. Cyanidin has the highest content in purple corn, reaching up to 1975 μg/g (Tian et al., 2022), while pelargonidin accounts for the highest proportion in red corn (Peniche-Pavía and Tiessen, 2020). Yellow corn is determined by carotenoids, with extremely low anthocyanin content (Sánchez-Nuño et al., 2024). During kernel development, anthocyanins can accumulate in large amounts and, due to their water-soluble nature, can even diffuse into tissues such as the endosperm. Because anthocyanins are inherently unstable, free forms are rarely present under natural conditions. Nucleotide sugar donors, including UDP-glucose, UDP-galactose, and UDP-xylose (Wang et al., 2018), serve as substrates in glycosylation reactions with the hydroxyl groups of anthocyanin molecules, forming glycosylated anthocyanin derivatives. These glycosylated anthocyanins can further undergo esterification with aromatic or aliphatic acyl donors, ultimately forming more complex and stable acylated anthocyanins. In plants, anthocyanins are mainly stored in vacuoles in their glycoside forms, such as cyanidin-3-O-glucoside and pelargonidin-3-O-glucoside. Anthocyanins exhibit a wide range of physiological functions and occur in various forms, including multiple types of anthocyanin glycosides. Their levels and composition change depending on the grain’s developmental stage and environmental conditions. Anthocyanins are highly sensitive to pH: in acidic environments (pH < 3), they maintain a stable red color, while in neutral or alkaline conditions (pH > 7), they degrade quickly, causing color changes. Factors such as UV radiation (Shi et al., 2023), high temperatures (Zou et al., 2023), and oxidases can accelerate their degradation, leading to the fading of coloration. Certain metal ions, like iron and copper, can interact with anthocyanins and cause discoloration or instability. Conversely, antioxidants, such as vitamins C and E, can, to some extent, protect anthocyanins from oxidative damage.

## 3 Structural genes involved in anthocyanin biosynthesis

**Figure 1:**
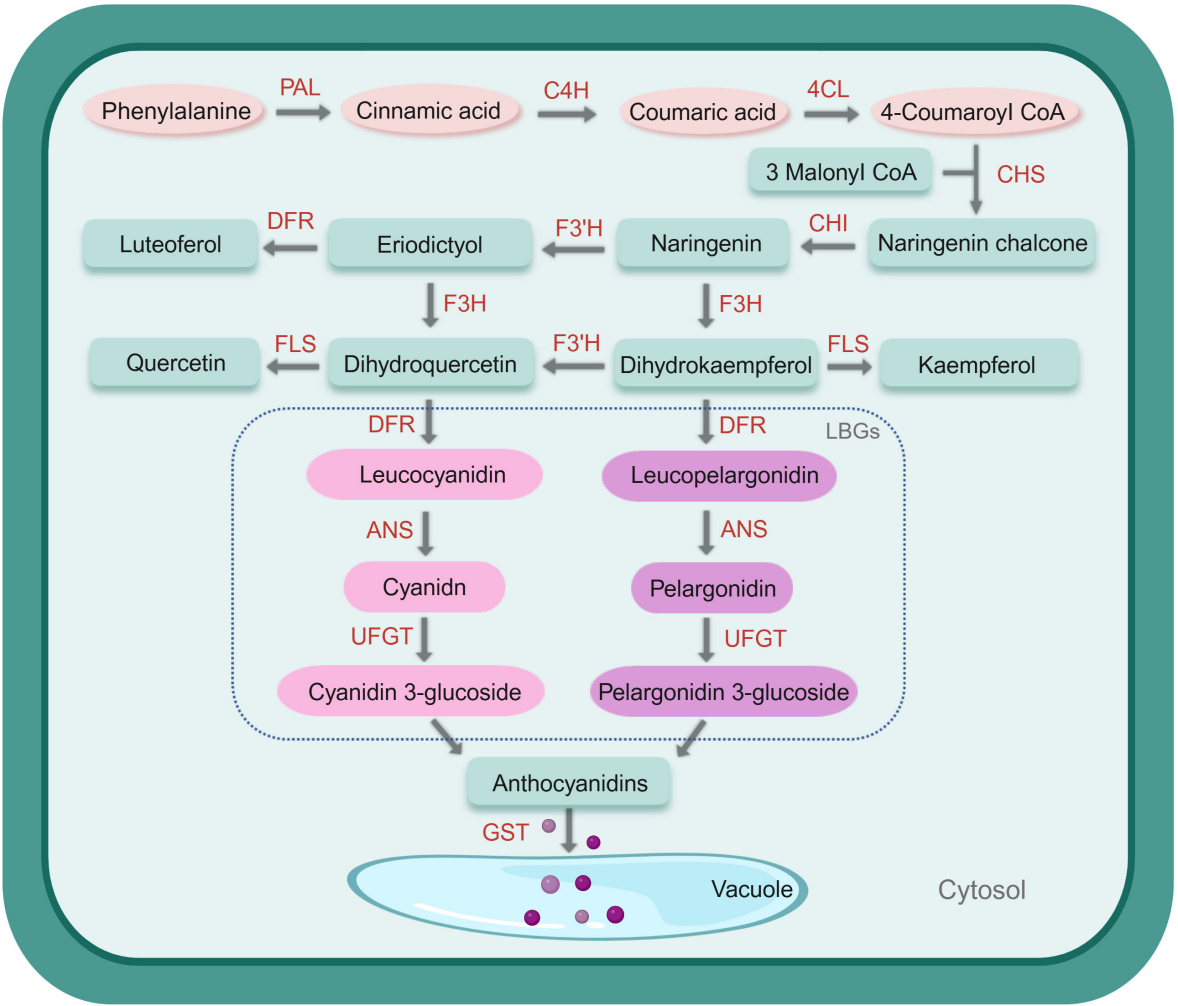
Anthocyanin biosynthetic pathway in maize. Schematic representation of the anthocyanin biosynthetic pathway in maize. Key enzymes and intermediates are shown, with structural genes highlighted in red. Created with BioRender.com.

The biosynthesis of anthocyanins is a key process in plant secondary metabolism. In maize, this process begins with the amino acid phenylalanine, which acts as the initial precursor. A series of coordinated enzymatic reactions, controlled by multiple genes, convert phenylalanine into anthocyanins (**Figure 1**). During these reactions in the cytoplasm, anthocyanins undergo multiple-site modifications, including hydroxylation, glycosylation, methylation, and acylation. Specialized transport proteins then transport the resulting anthocyanins into the vacuole, where they are stored in the form of colored aggregates (Goodman et al., 2004; Lago et al., 2013). Anthocyanins are the end products of a specialized branch of the flavonoid biosynthetic pathway. The pathway begins with phenylalanine, which is deaminated by the enzyme phenylalanine ammonia-lyase (PAL) to form cinnamic acid. Next, cinnamic 4-hydroxylase (C4H) introduces a hydroxyl group at the para position of the benzene ring, producing p-coumaric acid. This compound is then activated by 4-coumaroyl-CoA ligase (4CL) through adenylation and thioesterification reactions, resulting in the formation of p-coumaroyl-CoA (Mora et al., 2022). Secondly, chalcone synthase (CHS) catalyzes the condensation of p-coumaroyl-CoA with three molecules of malonyl-CoA to form naringenin chalcone. This reaction is a crucial early step in the flavonoid biosynthetic pathway and is generally considered the rate-limiting step of the process (Dixon et al., 2002). Following this, naringenin chalcone undergoes stereospecific isomerization by chalcone isomerase (CHI), producing naringenin, a colorless flavanone. Naringenin is further hydroxylated by flavanone 3-hydroxylase (F3H) to generate dihydrokaempferol (DHK). Using either naringenin or DHK as a substrate, flavonoid 3’-hydroxylase (F3’H) adds a hydroxyl group at the 3’-position of DHK, forming dihydroquercetin (DHQ). This intermediate can then be further hydroxylated by flavonoid 3’,5’-hydroxylase (F3’5’H) to produce dihydromyricetin (DHM). These reactions represent the core steps in flavonoid biosynthesis (Zhang et al., 2018). Among the enzymes involved, CHS and CHI are recognized as key rate-limiting enzymes in the anthocyanin biosynthetic pathway. Subsequently, dihydroflavonol 4-reductase (DFR) catalyzes the sequential reduction of DHK, DHQ, and dihydromyricetin (DHM) into colorless leucopelargonidin, leucocyanidin, and leucodelphinidin, respectively (Liu et al., 2021). These anthocyanidins then act as substrates for anthocyanidin synthase (ANS) and, under the action of flavonoid 3-O-glucosyltransferase (UFGT), they undergo glycosylation to form pelargonidin-3-glucoside, cyanidin-3-glucoside, and delphinidin-3-glucoside (Peniche-Pavía et al., 2022). These anthocyanin glucosides can then undergo further modifications, including acylation and methylation, catalyzed by enzymes such as anthocyanin acyltransferase (AAT), resulting in the formation of stable anthocyanins (Alappat and Alappat, 2020; Marin-Recinos and Pucker, 2024). Finally, glutathione S-transferase (GST) mediates the conjugation of glutathione with cyanidin 3-glucoside (C3G), and the resulting glutathionylated C3G is then directionally transported into the vacuole via the glutathione pump (GS-X pump) (Alfenito et al., 1998), where stable anthocyanins accumulate.

The structural genes directly encode the enzymes necessary for the anthocyanin biosynthetic pathway (Wang et al., 2025b). Among these, CHS, CHI, F3H, and F3’H are the key enzymes involved in the early, preparatory stages of anthocyanin synthesis, whereas DFR, ANS, and UFGT function in the later stages of the pathway (**Table 1**). CHS serves as the first rate-limiting enzyme in anthocyanin biosynthesis (Zhu and Zhou, 2023). Genome-wide analyses in maize have identified 15 CHS family members, with C2 and Whp characterized as the principal chalcone synthases. Both genes are expressed across multiple tissues and developmental stages in maize (Han et al., 2016). Studies have shown that mutations at critical catalytic sites in *CHS* genes can disrupt their conserved β-fold conformation, leading to enzyme inactivation. For example, in *ZmC2*, the substitution of the conserved glutamic acid at position 183 with lysine (E183K) causes the aggregation of the protein. This mutation impairs the enzyme’s ability to catalyze the initial reaction of the flavonoid biosynthetic pathway, ultimately resulting in maize kernels with a colorless phenotype (Della Vedova et al., 2005). Chalcone isomerase (CHI) is the first enzyme identified in the flavonoid biosynthesis pathway and serves as the second rate-limiting enzyme in anthocyanin production. *CHI* genes have been successfully cloned from multiple plant species, such as *Zea mays*, *Arabidopsis thaliana*, and *Morus alba* (Chao et al., 2021; Grotewold and Peterson, 1994; Jiang et al., 2015). Researches have shown that the *ZmF3’H* gene is a direct downstream target of the regulatory gene *pr1* (*Pericarp color1*) (Larson et al., 1986). The type of pigment produced depends on the *pr1* allele: when the allele is homozygous recessive, red pelargonidin pigments are synthesized, whereas a homozygous dominant *Pr1* allele leads to the production of purple cyanidin pigments (Peniche-Pavía et al., 2022). Dihydroflavonol 4-reductase (DFR) is a key enzyme in the biosynthesis of anthocyanins. The gene encoding DFR was first successfully isolated from maize in 1985 via transposon tagging and designated as *ZmA1* (O’Reilly et al., 1985). Functional studies confirmed its role in anthocyanin production: when the *ZmA1* cDNA sequence was fused to the constitutive CaMV 35S promoter and introduced into an anthocyanin-deficient mutant, the resulting transgenic plants exhibited a marked brick-red pigmentation, demonstrating that DFR is essential for anthocyanin biosynthesis (Haselmair-Gosch et al., 2018). Another critical enzyme in this pathway is ANS, a dioxygenase that catalyzes the final step of anthocyanin production by converting colorless leucoanthocyanidins into colored anthocyanidins. In maize, the *a2* gene represents the only known *ANS* gene and was first isolated in 1990 through transposon tagging (Menssen et al., 1990). The *a2* gene enables the conversion of colorless anthocyanidins into pigmented anthocyanins in the *A2* mutants of maize. Mutations in the *a2* gene disrupt anthocyanin accumulation, leading to changes in plant coloration. For instance, in onion (*Allium cepa*), mutations in the *ANS* gene cause the pericarp color to change from purple to yellow (Kim et al., 2005).

**Table 1:** Anthocyanin biosynthetic genes.

Gene name	Gene number	Location	Direction	Fragment size	Function
pal (PAL)	GRMZM2G074604	Chr5	←	3741	Phenylalanine lyase
c4h (C4H)	GRMZM2G140817	Chr6	→	4464	Cinnamic acid hydroxylase
bm5 (4CL)	GRMZM2G075333	Chr5	←	4586	Coumaric acid COA ligase
c2 (CHS)	GRMZM2G422750	Chr4	→	3397	Chalcone synthase
chi1 (CHI)	GRMZM2G155329	Chr1	←	1528	Chalcone isomerase
fht1 (F3H)	GRMZM2G062396	Chr2	→	1943	Flavanone 3- hydroxylase
pr1 (F3’H)	GRMZM2G025832	Chr5	←	2070	Flavonoid 3’- hydroxylase
fls1 (FLS1)	GRMZM2G152801	Chr5	←	1481	Flavonol synthase
fls2 (FLS2)	GRMZM2G069298	Chr5	←	1333	Flavonol synthase
a1 (DFR)	GRMZM2G026930	Chr3	←	1834	Dihydroquercetin reductase
a2 (ANS)	GRMZM2G345717	Chr5	→	2838	Cryptanthocyanin dioxygenase
bz1 (UFGT)	GRMZM2G165390	Chr9	←	1858	UDP glucose flavonoid 3-O glycosyltransferase
bz2 (GST)	GRMZM2G016241	Chr1	←	1002	24kD glutathione S transferase

Key structural genes involved in anthocyanin biosynthesis in maize, including gene IDs, genomic locations, and functional annotations.

## 4 Kernels transcriptional regulation governing anthocyanin biosynthesis

Structural genes encode the enzymes required for anthocyanin biosynthesis, but their expression is tightly regulated in a spatiotemporal manner by a suite of TFs. Central to this regulation is the MYB-bHLH-WD40 (MBW) complex, which acts as a key transcriptional regulator. The structural genes encode a series of enzymes that catalyze the sequential reactions of the anthocyanin biosynthetic pathway, while their transcription is governed by multilayered transcriptional regulatory networks. Three main families of TFs-MYB, basic helix-loop-helix (bHLH), and WD40 repeat proteins-interact to form the MBW ternary complex (**Table 2**). This complex specifically binds to the promoter regions of multiple structural genes involved in the anthocyanin biosynthetic pathway, precisely modulating their transcriptional activity. Through this regulatory mechanism, the MBW complex governs flavonoid accumulation and ultimately influences anthocyanin biosynthesis and deposition in plant tissues (Cao et al., 2018).

**Table 2:** Anthocyanin biosynthesis regulatory genes.

Gene name	Gene number	Location	Direction	Fragment size	Function
c1	GRMZM2G005066	Chr9	←	1073	R2R3-MYB
pl1	GRMZM2G701063	Chr6	→	1081	R2R3-MYB
p1	GRMZM2G084799	Chr1	→	10550	MYB
r1	GRMZM5G822829	Chr10	→	9268	bHLH
b1	GRMZM2G172795	Chr2	→	4798	bHLH
in1	GRMZM2G042733	Chr7	→	6277	bHLH
pac1	GRMZM2G058432	Chr5	←	3511	WD40

Key regulatory genes involved in anthocyanin biosynthesis in maize, including gene IDs, genomic locations, and functional annotations.

In the MBW complex, MYB TFs function as the primary determinants of its regulatory function and are prevalent across all eukaryotes. They are characterized by a highly conserved N-terminal DNA-binding domain (Tan et al., 2020), which typically contains one to four imperfect repeat sequences (R1, R2, R3, and R4). Based on this core structural feature, MYB TFs are classified into four subfamilies: 1R-MYB, R2R3-MYB, R1R2R3-MYB, and 4R-MYB. Among these, R2R3-MYB proteins are the most abundant in plants (Duan et al., 2022). They can specifically bind to target DNA sequences, enabling precise regulation of the transcription of structural genes involved in the anthocyanin biosynthetic pathway. In plants such as *Arabidopsis thaliana* and *Salvia miltiorrhiza*, studies have demonstrated that multiple MYB TFs—including TT2, MYB75, and MYB90—participate in the regulation of anthocyanin production in fruit tissues (Jaakola, 2013; Li et al., 2023). These TFs primarily function by activating the expression of downstream structural genes. For example, in strawberry, the R2R3-MYB TF FaMYB5 enhances anthocyanin biosynthesis by forming an MBW complex through interactions with FaEGL3 and FaLWD1/FaLWD1-like proteins (Jiang et al., 2023). Conversely, in *Fagopyrum tataricum*, the R2R3-MYB TF FtMYB3 functions as a transcriptional repressor, inhibiting anthocyanin biosynthesis by markedly downregulating the expression of key structural genes such as *DFR*, *ANS*, *BAN*, and *TT13* (Wang et al., 2022a). Members of the R3-MYB subfamily generally function as negative regulators of anthocyanin biosynthesis. In maize, a recently identified R3-MYB family member, Mybr97, was found to act as an important negative regulator that fine-tunes the anthocyanin biosynthetic pathway (Paulsmeyer and Juvik, 2023). Two well-characterized MYB TFs in maize, *c1* (*colored aleurone1*) and *pl1* (*purple plant1*), positively regulate anthocyanin biosynthesis by activating anthocyanin biosynthetic genes in distinct tissues. *C1* primarily functions in the endosperm and aleurone layers of maize kernels, where it serves as a core regulatory component of anthocyanin biosynthesis. Heterologous expression studies have demonstrated that introducing *ZmC1* into wheat induces significant pigment accumulation in vegetative tissues such as the coleoptile and stem (Riaz et al., 2019). A homologous gene, *Zmpl*, which shares high sequence similarity with *ZmC1*, has also been isolated from maize (Cone et al., 1993). The *pl* gene not only regulates pigmentation in floral and vegetative organs but also modulates anthocyanin biosynthesis by activating or repressing the expression of several key structural genes in the flavonoid pathway.

The bHLH proteins are key TFs in plants, characterized by two evolutionarily conserved and functionally distinct regions. The C-terminal α-helix–loop–α-helix (HLH) domain enables dimerization and binding to the promoter regions of target genes, thereby regulating gene expression (Ortolan et al., 2024). Meanwhile, the N-terminal basic amino acid region specifically interacts with DNA cis-acting elements (Feller et al., 2011). In the anthocyanin biosynthetic pathway, bHLH TFs play a key regulatory role in controlling structural gene expression (Goff et al., 1992). The *Lc* gene in maize was the first member of the bHLH family to be successfully isolated and molecularly cloned (Li et al., 2022a). Two homologous genes of *Lc*, namely *R1* (*Seed color component at R1*) and *B1* (*Colored plant 1*), have also been identified in maize. The *R1* gene is predominantly active in the pericarp, whereas *B1* functions mainly in the aleurone layer (Radicella et al., 1992). Studies have demonstrated that allelic variation in the *ZmR1* gene significantly affects anthocyanin accumulation in different tissues of maize. For example, the *ZmR1^CQ01^* cDNA, cloned from the midrib of the maize inbred line CQ01, when overexpressed, induced a pronounced purple pigmentation throughout the plant and substantially increased anthocyanin levels in multiple tissues. This approach enabled the development of a high-anthocyanin purple maize hybrid, Jingke 968 (Luo et al., 2022). In addition, functional molecular markers based on the *ZmR1* gene sequence have been developed, providing efficient tools for precise enhancement of anthocyanin content and for marker-assisted selection in maize breeding programs. Furthermore, researchers introduced the maize TFs *ZmC1* (MYB) and *ZmR1* (bHLH) into wheat via *Agrobacterium*-mediated transformation, generating transgenic wheat lines with increased anthocyanin content (Riaz et al., 2019). The results demonstrated that co-expression of *ZmC1* and *ZmR1* effectively increased anthocyanin accumulation through transcriptional regulation (Jian et al., 2019).

WD40 proteins do not directly recognize the promoters of target genes; rather, they function as co-regulators that enhance gene activation (Tian et al., 2024). They primarily mediate covalent histone modifications and participate in the dynamic remodeling of chromatin architecture, thereby indirectly modulating the transcriptional activity of downstream genes. As scaffold components of the MBW complex, WD40 proteins facilitate interactions between MYB and bHLH TFs, stabilizing the assembly of the complex. The maize TF *pac1* (*pale aleurone color1*) was the first WD40 family member identified in plants. Studies have documented that *pac1* plays a crucial regulatory role in the anthocyanin biosynthetic pathway by directly activating the expression of related structural genes. Its activity occurs independently of other regulatory factors within this pathway (Selinger and Chandler, 1999).

In maize, the TFs MYB, bHLH, and WD40 cooperatively form the MBW ternary complex, which regulates anthocyanin biosynthesis. In this complex, the MYB TF binds specifically to DNA, the bHLH TF enhances transcriptional activity, and the WD40 protein functions as a scaffold to stabilize the complex. This synergistic interaction ensures precise control of anthocyanin biosynthesis. Among the MYB TFs, *c1* and *pl1* regulate anthocyanin biosynthesis with distinct tissue-specific expression patterns: *c1* is expressed exclusively in seeds, whereas *pl1* is expressed in vegetative tissues (Cappellini et al., 2021). These MYB TFs control anthocyanin biosynthesis by regulating the transcription of key structural genes in the biosynthetic pathway, both during development and in response to light. The *r1* and *b1* genes encode the bHLH proteins, and *pac1* encodes a WD40 protein. The MBW complex regulates the expression of key genes involved in anthocyanin biosynthesis, such as *CHS*, *DFR*, *ANS*, and *UFGT*, by directly binding to their promoter regions. Within the complex, MYB TFs recognize MBS elements in the promoters to target specific genes, bHLH TFs bind to E-box elements (CANNTG) to enhance transcriptional activity (Wang et al., 2022b), and WD40 proteins stabilize the complex, facilitating efficient activation of the target gene. In response to environmental cues such as light (Tian et al., 2019), hormones, or abiotic stress, the expression of MYB and bHLH TFs is upregulated, enabling them to assemble with WD40 proteins to form the functional MBW complex. This complex subsequently activates downstream structural genes in the anthocyanin biosynthetic pathway, ultimately increasing anthocyanin accumulation in maize tissues.

## 5 Transcriptional regulation of the anthocyanin biosynthetic pathway

Beyond the characterization of regulatory genes, understanding the coordination of transcriptional control is essential for elucidating the regulatory hierarchy of the anthocyanin synthesis pathway. In this pathway, transcriptional regulation is a major determinant of anthocyanin production. Specific TFs precisely direct anthocyanin biosynthesis by recognizing and binding to cis-regulatory elements located upstream of structural genes (Busche et al., 2023). Among these regulatory factors, the MYB, bHLH, and WD40 protein families are particularly important. They can function individually or form binary or ternary complexes to positively or negatively regulate anthocyanin biosynthesis (An et al., 2012). Notably, the MBW complex governs the activation of late-stage biosynthetic genes in the flavonoid pathway and exhibits substantially higher transcriptional activation efficiency than individual TFs. The MBW complex exhibits markedly different functional modes across plant species. In Arabidopsis thaliana, the MBW complex is highly diversified, with multiple MYB TFs (PAP1/PAP2/MYB113/MYB114) forming distinct complexes with bHLH TFs (TT8/GL3/EGL3) and the WD40 TFs (TTG1) to regulate anthocyanin biosynthesis as well as epidermal cell development (Miller et al., 2015), including root hair and trichome formation (Zumajo-Cardona et al., 2023). By contrast, maize predominantly employs a core MBW complex composed of MYB TFs (C1/Pl1), bHLH TFs (R1/B1), and WD40 TFs (PAC1), which confers relatively centralized and efficient control of anthocyanin structural genes rather than multiple branched regulatory modules (Luo et al., 2025). In rice, the core components MYB TFs (C1), bHLH TFs (S1), and WD40 TFs (WA1) display a clear hierarchical expression pattern, in which TF S1 acts as an upstream regulatory node to sequentially activate TF C1 and TF WA1, thereby inducing anthocyanin biosynthetic genes (Sun et al., 2022). This shift from a complex, branched regulatory architecture to a simplified, hierarchical mode likely reflects evolutionary functional reorganization of the MBW complex and provides a conceptual basis for implementing branch-specific or hierarchical regulatory strategies to enable precise manipulation of anthocyanin accumulation in maize. Spelt et al. reported that the ectopic expression of the MYB family member *PhAN2* in petunia leaves significantly activates the transcription of the bHLH TF *PhAN1* (Spelt et al., 2000). Similarly, in anther tissue, the MYB homolog *PhAN4* also activates *PhAN1* transcription, suggesting that both factors act as upstream positive regulators of *PhAN1*. Further research revealed that PhAN2 and PhAN4 can interact with the WD40 protein PhAN11 to form an MBW ternary complex with transcriptional activation activity. This complex directly regulates the expression of *DFR*, a key structural gene in the anthocyanin biosynthesis pathway, thereby initiating anthocyanin production. In contrast, *PhMYB27* functions as a negative regulator in petunia; by repressing *PhAN1* expression, it indirectly inhibits the transcriptional activity of multiple anthocyanin biosynthesis-related genes, leading to reduced anthocyanin accumulation (Albert et al., 2014). In tomato, the bHLH TF *SlJAF13* functions as a positive regulator of anthocyanin biosynthesis, promoting anthocyanin accumulation in fruits by upregulating the expression of *SlAN1* (Cappellini et al., 2021). SlJAF13 initially interacts with the MYB TF SlAN2-like and the WD40 TF SlAN11 to form a ternary complex. This complex specifically binds to the G-box cis-acting element in the promoter region of *SlAN1*, thereby activating its transcription. The upregulated expression of *SlAN1* subsequently enhances the expression of downstream structural genes, including *SlDFR*, collectively driving anthocyanin biosynthesis. Both MYB and bHLH TFs are capable of synergistically regulating anthocyanin biosynthesis without relying on WD40 proteins, directly controlling the expression of anthocyanin biosynthetic genes. For instance, in radish, the interaction between *RsTT8* and *RsMYB1* strongly activates the transcription of several anthocyanin biosynthetic genes, leading to increased anthocyanin accumulation (Lim et al., 2017). To clarify the molecular mechanism by which the MBW complex regulates anthocyanin production, Albert et al. proposed a detailed mechanistic model. According to this model, under non-inductive conditions, MYB repressors are highly expressed, while bHLH and WD40 proteins maintain constitutive expression. MYB repressors inhibit anthocyanin biosynthesis through two mechanisms: first, by directly binding to the promoters of anthocyanin biosynthetic genes and suppressing their transcription, and second, by competitively binding free intracellular bHLH proteins, thereby preventing the formation of the MBW ternary complex. This dual regulatory mechanism allows MYB repressors to effectively suppress anthocyanin production.

## 6 Signaling pathways involved in anthocyanin biosynthesis

In addition to being regulated at the transcriptional level, anthocyanin biosynthesis is strongly influenced by multiple signal transduction pathways. In maize, the accumulation of anthocyanins is governed by an intricate network of multiple signal transduction pathways. These pathways include light signaling, hormone signaling, sugar signaling, and various environmental cues. Among them, the light signaling pathway plays a particularly important role, modulating anthocyanin levels in maize kernels through sophisticated mechanisms that integrate signal transduction and gene expression control (Xiang et al., 2022). This process is influenced not only by factors such as light quality, light intensity, and photoperiod, but also by interactions among different photoreceptors and TFs. Photoreceptors are essential molecular components that detect changes in light and convert these signals into biochemical responses. Upon receiving light signals, photoreceptors trigger a cascade of downstream signaling events that induce or repress specific TFs, such as MYB, bHLH, and WD40 (He et al., 2019). These TFs directly control the expression of key genes in the anthocyanin biosynthetic pathway, thereby regulating anthocyanin production. For example, PhyA and PhyB modulate anthocyanin biosynthesis by influencing the ubiquitination activity of COP1 and the stability of HY5 (Kim et al., 2022). Similarly, the blue-light photoreceptors, CRYs, regulate the expression of anthocyanin-related biosynthetic genes by influencing the stability of PIFs (Yang and Liu, 2020). In maize, the regulation of anthocyanin biosynthesis by light occurs at the level of TFs (Chachar et al., 2024). Furthermore, plant hormones, including abscisic acid (ABA) (An et al., 2018), jasmonic acid (JA) (Wu et al., 2024), gibberellins (GA) (Zhang et al., 2017), and ethylene (ETH) (Liu et al., 2025), modulate anthocyanin biosynthesis by activating their respective signaling pathways. Extensive researches have demonstrated that ABA exerts a pronounced inductive effect on anthocyanin accumulation in numerous plant species. However, the detailed molecular mechanisms by which ABA regulates the anthocyanin biosynthetic pathway are not yet fully understood. The *VP1* gene plays an essential role in maize seed maturation, and loss-of-function mutations in *VP1* render seeds unresponsive to ABA, thereby suppressing the expression of the downstream anthocyanin regulatory gene *C1*, ultimately impairing anthocyanin biosynthesis (McCarty et al., 1989). JA is another crucial plant hormone that regulates anthocyanin accumulation through JAZ proteins. These proteins repress anthocyanin accumulation primarily by directly interacting with the R2R3-MYB TFs (PAP1, PAP2) and bHLH TFs (GL3, EGL3, TT8) within the MBW complex (Qi et al., 2011). Additionally, JAZ proteins can bind to other bHLH factors to alleviate their inhibitory effects on anthocyanin production. This dual function highlights the complex role of JAZ proteins in the anthocyanin regulatory network. GA can indirectly influence anthocyanin biosynthesis by modulating the stability of DELLA proteins. DELLA proteins interact with transcriptional repressors, such as MYBL2 and JAZ, which inhibit anthocyanin biosynthesis, as well as with positive regulators, such as PAP1/MYB75 (Wan et al., 2024), which enhance the activity of the MBW complex and promote anthocyanin biosynthesis. In contrast, GA promotes the degradation of DELLA proteins, reducing their regulatory influence and thereby suppressing DELLA-mediated anthocyanin biosynthesis (Xie et al., 2016). Anthocyanin accumulation in plants is controlled by both positive and negative regulators. Studies on the *Arabidopsis icx1* mutant have demonstrated that loss of *ICX1* function results in pronounced upregulation of *CHS* and multiple flavonoid biosynthetic genes under a range of environmental conditions. These findings establish *ICX1* as a key negative regulator that operates through a complex network of light-signaling pathways. Specifically, *ICX1* modulates light-dependent responses mediated by UVR8, CRY1, and PhyA, as well as non-light signaling pathways responsive to low temperature, sucrose, and cytokinins. Collectively, these pathways act in concert to directly or indirectly repress *CHS* expression and anthocyanin biosynthesis (Wade et al., 2003). Sugar-signaling pathways play an important role in regulating anthocyanin accumulation. For instance, loss of microRNA828 (miR828) function reduces sucrose-induced anthocyanin accumulation, while plants overexpressing miR828 exhibit lower anthocyanin levels than wild-type plants in response to sucrose (Zhang et al., 2020). External environmental cues modulate the expression of genes associated with the anthocyanin biosynthetic pathway, thereby influencing both the synthesis and accumulation of anthocyanins and maintaining their homeostasis within plants. These environmental factors influence the expression of related genes to regulate anthocyanin synthesis and accumulation, ensuring a dynamic equilibrium of anthocyanin levels in plant tissues. Researches indicate that nitrogen application has a significant effect on anthocyanin accumulation in the kernels of purple glutinous maize. An optimal nitrogen supply enhances the activity of enzymes involved in anthocyanin biosynthesis, leading to increased pigment accumulation (Feng et al., 2024).

**Figure 2:**
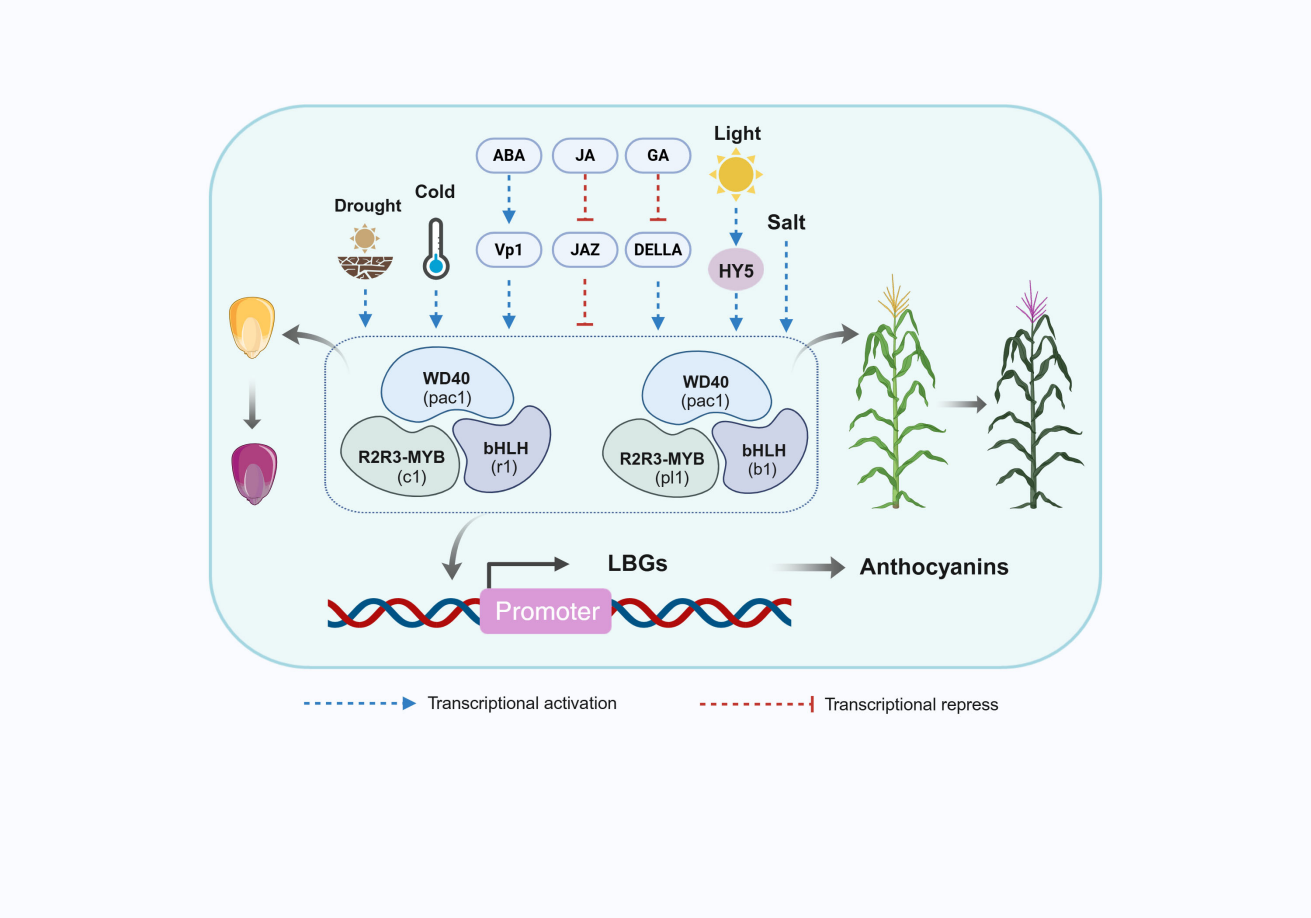
Molecular regulatory network governing anthocyanin accumulation in maize. Environmental factors (drought, cold, light, and salt) enhance MBW complex expression, with light modulating anthocyanin biosynthesis through HY5 stability. VP1 mutation reduces ABA sensitivity in seeds and alters anthocyanin production. JAZ proteins in the JA signaling pathway suppress anthocyanin accumulation, while GAs indirectly affect its synthesis by regulating DELLA stability. ABA and JA upregulate MBW expression, whereas GAs repress its transcription. c1/pl1 encode R2R3-MYB proteins, r1/b1 encode bHLH proteins, and pac1 encodes a WD40 protein. Seed-expressed c1/r1 and vegetative tissue-specific pl1/b1 form MBW complexes that bind promoter regions of multiple anthocyanin biosynthetic genes to modulate their transcription. Created with BioRender.com.

The accumulation of anthocyanins in maize results from the coordinated regulation of multiple signal transduction pathways that modulate gene expression through the activation of key TFs, the assembly of regulatory complexes, and the integration of diverse signaling inputs (**Figure 2**). The dynamic interplay among light, hormonal, sugar, and environmental signaling pathways collectively governs the efficiency of anthocyanin biosynthesis and its overall accumulation.

## 7 Epigenetic regulation of anthocyanin biosynthesis

Apart from classical signaling pathways, anthocyanin biosynthesis is also regulated by complex epigenetic mechanisms that dynamically and precisely adjust gene expression in response to developmental cues and environmental stress. Epigenetic regulation involves heritable changes in gene expression that occur without altering the underlying DNA sequence (Osorio et al., 2013). These mechanisms include DNA methylation (Liu et al., 2023), covalent histone modifications (Jarillo et al., 2009), chromatin remodeling (Iyer, 2012), RNA-mediated regulation (Zhao et al., 2019), and changes in nucleosome positioning. Epigenetic variation represents a fundamental mechanism governing the regulation of anthocyanin metabolism. It contributes to heritable phenotypic variations in floral coloration, leaf pigmentation, pericarp color, and flesh coloration without alterations in the underlying DNA sequence. Instead, epigenetic modifications influence anthocyanin biosynthesis through the modulation of regulatory gene expression. Moreover, epigenetic variation may indirectly affect anthocyanin accumulation by influencing genome stability. Variation in DNA methylation can modulate the stability of gene expression by the addition of methyl groups to CpG islands or non-CpG sites (Deaton and Bird, 2011), thereby repressing gene transcription and consequently influencing plant growth, development, and adaptability. In radish, Wang et al. documented (Wang et al., 2020) that transposable elements contribute to promoter methylation. Notably, hypermethylation of a CACTA transposon facilitates the spread of DNA methylation into the promoter region of the key anthocyanin regulatory factor RsMYB1. This heritable methylation modification results in reduced transcription of RsMYB1, ultimately suppressing anthocyanin biosynthesis and giving rise to the white-fleshed mutant phenotype. Histone methylation is an extensively studied epigenetic modification, characterized by its diverse regulatory functions and numerous covalent modification sites. This modification predominantly occurs on arginine and lysine residues of histone proteins and modulates gene transcription through alterations in chromatin structure. In functional analyses of histone demethylases in poplar, Fan et al. discovered (Fan et al., 2018) that the H3K9-specific demethylase JMJ25 directly binds to the genomic locus of the negative regulator *MYB182*. Through chromatin demethylation at this locus, JMJ25 facilitates the transcriptional activation of *MYB182*, thereby suppressing the biosynthesis and accumulation of anthocyanins and proanthocyanidins. Researches in *Arabidopsis* have shown that ubiquitination negatively regulates anthocyanin accumulation by mediating the degradation of the principal anthocyanin biosynthetic regulators PAP1 and PAP2 (Maier et al., 2013). Within the epigenetic framework governing anthocyanin biosynthesis, DNA methylation typically suppresses gene expression, whereas histone modifications can function as either activators or repressors of transcription depending on their types and genomic contexts. Ubiquitination lowers anthocyanin levels by promoting the proteasome-mediated degradation of TFs (Skelly et al., 2019), while SUMOylation often counteracts this process, promoting protein stability and thereby supporting sustained gene expression (Khan and Abbas, 2023). Extensive crosstalk exists among these epigenetic mechanisms; for example, H3K9me2 and DNA methylation often display coordinated enrichment in plants (Du et al., 2012), underscoring the complexity and interconnectivity inherent to the epigenetic regulatory network.

## 8 Regulation of anthocyanin accumulation in response to abiotic and biotic stresses

In addition to epigenetic regulation, plants integrate multiple stress-responsive signals to fine-tune anthocyanin accumulation under both abiotic and biotic stresses. In maize kernels, adverse environmental factors such as low temperature, high salinity, and drought exert significant effects on anthocyanin accumulation. Anthocyanins, as a prominent class of secondary metabolites, not only contribute to pollinator attraction and seed dispersal but also enhance plant resilience to diverse biotic and environmental stresses. Under low-temperature stress, maize exhibits impaired root uptake of water and nutrients, accompanied by inhibition of chlorophyll biosynthesis. These physiological disruptions induce a phenotypic transition in leaves from green to purple, resulting in enhanced anthocyanin accumulation (Christie et al., 1994). Additionally, the combination of low-temperature and salt stress further elevates the activities of antioxidant enzymes in maize embryos, including superoxide dismutase (SOD), catalase (CAT), and ascorbate peroxidase (APX), suggesting that low-temperature stress may facilitate anthocyanin accumulation through activation of the antioxidant defense system (Wang et al., 2025a). In *Brassica napus*, overexpression of the *AtDFR* gene significantly upregulates the transcription of endogenous *DFR* genes, thereby promoting anthocyanin biosynthesis and accumulation, which in turn enhances salt tolerance (Kim et al., 2017). Conversely, mutants deficient in key genes involved in anthocyanin biosynthesis exhibit markedly reduced salt tolerance (Li et al., 2017). Drought stress exerts a significant influence on anthocyanin biosynthesis. Under drought conditions, photosynthetic activity in maize leaves declines, and the efficiency of photosystem II (PSII) is reduced, thereby limiting the accumulation of photosynthetic products (Li et al., 2021). Interestingly, drought stress can also stimulate anthocyanin biosynthesis in maize plants, which contributes to improved drought tolerance. For example, maize plants overexpressing the transgenic *TPS1* gene show higher anthocyanin levels under drought, likely because *TPS1* expression promotes both root growth and anthocyanin biosynthesis (Zhao et al., 2017). Furthermore, drought stress increases the activity of antioxidant enzymes, such as SOD, peroxidase (POD), and CAT in maize (Singh et al., 2023), thereby mitigating reactive oxygen species accumulation and protecting cellular membranes from damage. These findings indicate that abiotic stresses influence anthocyanin accumulation in maize kernels through several interconnected mechanisms, thereby facilitating plant survival and adaptation under adverse environmental conditions.

## 9 Future perspectives and strategic approaches for anthocyanin regulation in maize

The accumulation of anthocyanins in plants is influenced by a complex interplay of positive and negative regulatory factors. Previous studies have predominantly concentrated on positive regulators, whereas the mechanisms underlying negative regulation remain comparatively underexplored. The molecular processes involved in anthocyanin modification, vacuolar transport, and associated regulatory pathways, including those mediated by GST transport proteins and MATE transporters, remain to be fully explored. Additionally, the influence of epigenetic modifications on anthocyanin regulation in response to environmental cues requires further investigation. Future studies should aim to systematically elucidate the interactions between positive and negative regulators within anthocyanin regulatory networks, with particular emphasis on the functions of MYB repressors and bHLH inhibitors, to clarify their contributions to the spatiotemporal accumulation of anthocyanins. Future researches should leverage multi-omics approaches—including transcriptomics, metabolomics, and epigenomics—to unravel the spatiotemporal regulatory networks controlling anthocyanin biosynthesis. For instance, combined RNA-seq and metabolomic profiling across different developmental stages of maize kernels has revealed tightly coordinated temporal dynamics between anthocyanin structural genes, regulatory transcription factors, and flavonoid metabolites, demonstrating that anthocyanin accumulation is controlled by stage-specific transcriptional programs rather than constitutive pathway activation (Guo et al., 2024; Zhang et al., 2025). Importantly, emerging epigenomic analyses, including genome-wide DNA methylation mapping and ATAC-seq–based chromatin accessibility profiling, have uncovered dynamic epigenetic landscapes during maize seed development, in methylation changes are closely associated with the activation or repression of anthocyanin-related genes and regulatory loci (Li et al., 2025b; Xu et al., 2019). Classical epigenetic phenomena such as paramutation at the *r1*/*B1* locus further illustrate how heritable epigenetic states can modulate anthocyanin pigmentation independently of DNA sequence variation, highlighting an additional regulatory layer contributing to spatial and temporal variation in kernel coloration (Chandler and Stam, 2004). Together, these multi-omics approaches enable the identification of developmental stage–specific regulatory nodes, epigenetic switches, and metabolite–gene associations, thereby providing a mechanistic basis for translating fundamental regulatory insights into precision breeding strategies for enhancing anthocyanin content in maize kernels. In addition, targeted genome editing using CRISPR-Cas9 on key regulatory nodes (e.g., *ZmC1*, *ZmR1*, *ZmPr1*) offers immense potential for developing maize varieties with high anthocyanin content, improved stress tolerance, and greater nutritional value (Lu et al., 2023). By integrating modern analytical technologies with environmental regulation strategies, the breeding and industrial application of nutritionally fortified maize varieties can be accelerated. This approach not only offers new opportunities for the development of the fresh corn industry but also provides essential technological support for the advancement of modern agriculture.
